# Assessing Biophysical and Physiological Profiles of Scalp Seborrheic Dermatitis in the Thai Population

**DOI:** 10.1155/2019/5128376

**Published:** 2019-07-08

**Authors:** Poonkiat Suchonwanit, Korn Triyangkulsri, Monthanat Ploydaeng, Kanchana Leerunyakul

**Affiliations:** Division of Dermatology, Department of Medicine, Faculty of Medicine, Ramathibodi Hospital, Mahidol University, Thailand

## Abstract

**Background:**

Scalp seborrheic dermatitis (SD) is a common and chronic inflammatory skin disease which tends to recur over time. By measuring biophysical properties of the stratum corneum, many studies report abnormal biophysical profiles and their association in various dermatologic diseases. The aim of the study is to analyze the biophysical properties and skin barrier defects of scalp SD compared to healthy controls.

**Materials and Methods:**

This study is a cross-sectional study assessing the correlation of various biophysical and physiological profiles in scalp SD. Forty-two Thai participants with scalp SD were enrolled in the study and 40 healthy participants were also enrolled as the control group. Both SD and control group were subjected to a one-time biophysical and physiological properties' measurement of transepidermal water loss (TEWL), stratum corneum hydration (SCH), skin surface pH, skin surface lipid, and skin roughness.

**Results:**

The mean TEWL of lesional skin of SD cases were significantly higher than those of control group (*P*<0.05). Relating to high mean TEWL, the mean SCH was found to be significantly lower in SD cases (*P*<0.05). Skin surface lipid was also found to be significantly higher in SD group (*P*<0.05). However, there were no differences in skin surface pH (*P*=0.104) and roughness (*P*=0.308) between the two groups. Pairwise comparison of each subgroup found that moderate and severe SD demonstrated significantly higher mean skin surface lipid than that of control group (*P*<0.05).

**Conclusion:**

Scalp SD may be associated with seborrhea in Thai population. Monitoring of SCH, TEWL, and skin surface lipid could be helpful in assessing severity and evaluating the treatment outcome in patients with scalp SD.

## 1. Introduction

Seborrheic dermatitis (SD) is a common chronic recurring inflammatory skin disease located in seborrheic area. It affects 2.35%–11.3% of the general population [1]. Scalp is one of the common sites of involvement and if dandruff was included as having SD, the incidence of SD was found to be 10.16%–11.6% and as high as 20%–50% in some studies [2–6]. It has been widely hypothesized that three major factors, namely,* Malassezia* proliferation, increased sebum production, and individual predisposition, are involved in the development of SD. Recently, associations between scalp SD and scalp microbiome such as bacterial commensals and presence specific strains of* Malassezia* yeasts have been published. This information helps fill the gaps in the pathophysiology of scalp SD which is still not completely understood [7, 8].

The stratum corneum plays a crucial role as the skin barrier in regulating metabolic processes and functions. By measuring various biophysical properties of the stratum corneum, several studies report abnormal biophysical profiles and their association in various dermatologic diseases, such as atopic dermatitis (AD) and psoriasis. Compared to healthy population, various abnormal profiles such as increment in transepidermal water loss (TEWL) or reduction in stratum corneum hydration (SCH) were found in patients with these diseases [9–11]. As for SD, a critical factor in its pathogenesis may be the intrinsic quality of the stratum corneum. One study reported that the mean TEWL of neonatal patients with SD were relatively higher than that of the control group and the mean TEWL of these patients was reduced closer to the value of control group after treatment [12].

Scalp skin reveals a remarkable difference to the skin of other body regions. It comprises an abundance of hair follicles, sebaceous glands, and blood vessels, resulting in the dissimilarity of its biophysical properties compared to other parts of the body. To our knowledge, the biophysical profiles of scalp SD have never been investigated. Better understanding of their profiles may help establish the pathophysiology of the disease, assess the treatment efficacy, and improve the patient care. The aim of the study is to analyze the biophysical and physiological properties of scalp SD compared to healthy controls.

## 2. Materials and Methods

### 2.1. Study Design

This study is a cross-sectional study assessing the correlation of various biophysical profiles in scalp SD. The study was approved by the Committee of Human Rights Related to Research Involving Human Subjects, Mahidol University (ID03-59-19). The study adhered to the principles of the Declaration of Helsinki. All patients provided written and witnessed informed consent.

### 2.2. Participants

The sample size was estimated based on data from a previous study of TEWL and water content in the stratum corneum in SD [12]. To achieve a power of 80% and a level of significance of 5%, the calculated minimum number of subjects was 17 for each study group. Forty-two Thai participants with clinically diagnosed SD of the scalp were enrolled in the study and 40 healthy participants were also enrolled as the control group. We excluded the participants who had scalp and/or systemic diseases that impaired skin barrier function, e.g., AD, contact dermatitis, tinea capitis, and scalp psoriasis, neurological diseases mainly cerebrovascular diseases and Parkinson's disease, HIV infection, scalp conditions that might interfere with the measurement such as open wound on the measured site, and systemic and/or topical medications that alter the scalp properties.

### 2.3. Measurements

Both SD and control groups were subjected to a one-time biophysical and physiological properties' measurement. All participants were informed to avoid all types of hair and scalp products 12 hours prior to the examination. The measurement was performed by a well-trained and experienced investigator under standardized condition (temperature of 19°C–24°C and relative humidity of 50%–58%). All participants were in the measurement room for at least 15 minutes before the measurement for acclimatization.

Area with clinical evidence of SD on the scalp was chosen to be measured in SD group while occipital area of the scalp was chosen in control group. TEWL was measured using Tewameter® TM 300 (Courage + Khazaka electronic GmbH, Köln Germany), SCH was measured by Corneometer® CM 825 (Courage + Khazaka electronic GmbH, Köln Germany), skin surface pH was measured using Skin pH meter® PH 905 (Courage + Khazaka electronic GmbH, Köln Germany), skin surface lipid was measured by Sebumeter® SM 815 (Courage + Khazaka electronic GmbH, Köln Germany), and, lastly, skin roughness was assessed by Visioscan® VC 98 (Courage + Khazaka electronic GmbH, Köln Germany). Three readings of each measurement were made. All measurements were done on the same area and location. The mean values were then calculated from these readings.

For further analysis purpose, we asked a blinded dermatologist to categorize scalp SD cases into mild, moderate, and severe using 4-point scale scores which rate each scalp in terms of erythema (0 = no erythema, 1 = faint pink color, 2 = pink color, and 3 = red color), dandruff (0 = no dandruff, 1 = only scrapped, 2 = obvious scaling, and 3 = obvious sheets), and lesional extent (0 = no lesion, 1 = 1–30% of scalp area, 2 = 31–70% of scalp area, and 3 = 71–100% of scalp area). The sum of these components was obtained which classified each participant into mild (1–3), moderate (4–6), or severe SD (7–9) [13].

### 2.4. Statistical Analysis

Statistical analysis was performed using SPSS for Windows, version 18.0 (SPSS Inc., Chicago, IL, USA). The data were not analyzed separately for male and female participants as the preliminary analysis revealed no statistically significant difference in outcomes between genders. Categorical data, mainly sex, was expressed as proportions and was analyzed using chi-squared test. Continuous variables were expressed as mean ± standard deviation and the mean age was analyzed using t-test while measured variables were evaluated using analysis of variance with post hoc Tukey Honestly Significant Difference test. Disease duration among subgroups was compared using Kruskal–Wallis test. P value of less than 0.05 was considered to be statistically significant.

## 3. Results

The participants' demographic data is summarized in Table 1. The mean TEWL of lesional skin of scalp SD cases were significantly higher than those of control group (*P*<0.05). Relating to high mean TEWL, the mean SCH was found to be significantly lower in scalp SD cases (*P*<0.05). Skin surface lipid was also found to be significantly higher in scalp SD group (*P*<0.05). However, there was no difference in skin surface pH and roughness between the two groups (Table 2).

Through performing subgroup analysis, the mean skin surface lipid was found to be directly proportional with disease severity (*P*<0.05) (Table 3). Pairwise comparison of each subgroup found that moderate and severe scalp SD demonstrated significantly higher mean skin surface lipid than that of control group (*P*<0.05). The mean skin surface lipid was also lower in mild scalp SD cases when compared to moderate and severe cases but not significant (*P=*0.68 and* P*=0.53) (Table 4). Although the differences were apparent for other measurements, they failed to produce statistically significant difference from pairwise comparison (Table 3).

## 4. Discussion

Scalp SD and dandruff are considered as components of the same dermatological condition that affects the scalp where SD represents a more severe and less confined presentation. Despite being a relatively common condition, the pathogenesis of SD or dandruff is still unclear [14]. However, there is a general consensus establishing three main factors as the pivotal predispositions: first, the* Malassezia* yeasts, mainly* M. restricta* and* M. globosa *[15, 16]; second, the sebum level; and, third, individual susceptibility which possibly linked to the stratum corneum barrier abnormality [11]. The present study reports a significant increase of TEWL and skin surface lipid and a decrease of SCH, suggesting impaired skin barrier function in scalp SD patients.

The stratum corneum is a multilayered structure built with anucleated, flattened corneocytes which are linked by continuous specialized lipidic matrix [17]. Varying characteristic, composition, and organization of these constituents in stratum corneum of different body sites result in dissimilarity of permeability and cohesiveness of the skin in different body parts [18]. Therefore, biophysical properties of one area might not justify the whole body and values of each property might also differ greatly in each body site. Thus, scalp measurement should customarily be done in scalp-related conditions.

Among these miscellaneous elements of stratum corneum, corneodesmosome, a specialized intercellular protein that locks the corneocytes together, plays the most crucial role in maintaining the integrity of the stratum corneum [19, 20]. Skin hydration and pH were found to facilitate the hydrolytic enzymes that act on these corneodesmosome and influence the stratum corneum permeability barrier homeostasis and integrity [21]. In terms of increased sebum production, it was found to disturb the desquamation process and also impair lipid organization of the stratum corneum [22].

Scalp SD patients had increased TEWL similar to AD indicating impaired permeability function. Similar to AD, SCH of scalp SD was also found to be decreased as well [23–26]. In AD model, various degrees of inflammation causing filaggrin deficiency and ultimately resulting in insufficient natural moisturizing factor of the epidermis correlate with the diminishing value of SCH measured [27]. However, there is still no evidence linking SD to filaggrin abnormality. Possible explanation for abnormal TEWL and SCH in scalp SD is more likely to be the altered epidermal tight junction barrier function from the process of inflammation of the skin. This phenomenon was observed in dermatitis mice model and was found to be exclusive from filaggrin abnormality [28]. Severity of scalp SD, though not statistically significant in our study, also seemed to influence the degree of TEWL and SCH abnormalities with higher severity showing higher TEWL and lower SCH. Larger study population might be able to further justify these findings.

Interestingly, the scalp SD disease severity was found to have significant positive correlation with the mean skin surface lipid in our study. More severe disease was associated with higher mean skin surface lipid. Thus, our study supports the concept of seborrhea as an etiology of SD contradicting previous studies which were inconclusive or suggesting otherwise [29–32]. Sebocyte proliferation and sebum production were found to be under hormonal control which increases or decreases according to sebaceous gland biology [33, 34]. The bimodal distribution in the prevalence of SD, at birth and postpuberty, also suggests that SD may be related to male sex hormone activity [35–37]. A study on seborrhea in Parkinson's disease patients also demonstrated a higher prevalence of SD in both normal and parkinsonian male subjects compared to female subjects further supporting the role of androgen in sebum excretion and SD [38]. Increment in skin surface lipid was found to promote the growth of* Malassezia *yeasts while reduction in sebum production after isotretinoin treatment also impaired the growth of* Malassezia *yeasts and improved the severity of SD [39]. In addition to increased sebum production, alteration of lipid composition may also play a role in SD pathogenesis. Free fatty acid was lower and triglyceride was higher in both HIV and non-HIV patients with SD compared to patients without SD while free cholesterol, wax esters, and squalene showed no significant difference [40]. A study in India noted similar trends of having higher mean TEWL and surface lipid in patients with dandruff compared to healthy population. Compared to our study, the mean TEWL in the study was higher in both control and dandruff patients suggesting the effect of different population characteristics on the scalp biophysical profile [8].

The main limitation of our study is the small number of study population and being a single-center study. Furthermore, numerous influencing factors, such as sex, age, and ethnicity, may affect a standardized range or cut-off point of the biophysical and physiological parameters of scalp SD [41]. Thus, our subjects may not represent the general population. A well-planned study in a large number of participants is recommended. Another limitation of our study is that control patients were measured only on the occipital area while scalp SD patients were measured on their respective regional area. As different scalp regions may exhibit different structures and biophysical and physiological profiles, these differences may affect the results in our study.

## 5. Conclusion

In conclusion, Scalp SD may be associated with seborrhea or abnormality in skin sebum properties. Monitoring of SCH, TEWL, and skin surface lipid could be helpful in assessing severity and evaluating the treatment outcome of scalp SD.

## Figures and Tables

**Table 1:** Demographic data.

Variables	Case (n = 42)	Control (n = 40)	P value
*Age*, years			
mean ± SD	34.5 ± 6.9	32.4 ± 6.6	0.16
*Sex*, n (%)			
Male	15 (35.7%)	12 (30.0%)	0.58
Female	27 (64.3%)	28 (70.0%)	
*Duration of disease*, years			
median (range)	4 (1-11)	-	NA
*Measurement areas*, n (%)			
Frontal	4	0	NA
Vertex	9	0	
Parietal	15	0	
Occipital	14	40	

*NA*, not applicable.

**Table 2:** Biophysical properties of scalp seborrheic dermatitis cases and control.

Variables	Case	Control	95% CI	P value
	(n = 42)	(n = 40)		
*TEWL*, g/h/m^2^				
mean ± SD	13.91 ± 4.52	11.66 ± 3.93	0.44 - 4.15	<0.05*∗*
*SCH*, units				
mean ± SD	5.61 ± 1.24	6.42 ± 1.92	-1.49 - -0.10	<0.05*∗*
*pH*				
mean ± SD	4.95 ± 0.51	5.19 ± 0.62	-0.44 - 0.04	0.104
*Skin surface lipid*, units				
mean ± SD	35.63 ± 7.59	29.56 ± 6.35	3.04 - 9.15	<0.05*∗*
*Roughness*, units				
mean ± SD	11.55 ± 3.56	10.85 ± 2.61	-0.66 - 2.06	0.308

*SCH*, stratum corneum hydration;* TEWL*, transepidermal water loss. *∗*: statistically significant.

**Table 3:** Biophysical properties among various severity of scalp seborrheic dermatitis cases and control.

Variables	Mild (n = 14)	Moderate (n = 16)	Severe (n = 12)	Control (n = 40)	P value
*Age*, years					
mean ± SD	33.5 (5.5)	36.1 (6.9)	34.9 (6.4)	32.4 (6.6)	0.24
*Sex*					
male:female	6:8	5:11	4:8	12:28	0.84
*Duration of disease*, years					
median (range)	4 (1-10)	3.5 (1-11)	4 (1-9)	-	0.96
*TEWL*, g/h/m^2^					
mean ± SD	13.52 ± 4.65	13.89 ± 4.91	14.25 ± 5.24	11.66 ± 3.93	0.16
*SCH*, units					
mean ± SD	6.25 ± 1.24	5.91 ± 1.19	5.46 ± 1.25	6.42 ± 1.92	0.25
*pH*					
mean ± SD	4.92 ± 0.46	5.05 ± 0.65	4.94 ± 0.51	5.19 ± 0.62	0.56
*Skin surface lipid*, units					
mean ± SD	32.41 ± 6.52	35.19 ± 7.95	35.93 ± 6.99	29.56 ± 6.35	<0.05*∗*
*Roughness*, units					
mean ± SD	11.26 ± 3.25	11.91 ± 3.65	11.46 ± 3.59	10.85 ± 2.61	0.66

*SCH*, stratum corneum hydration;* TEWL*, transepidermal water loss. *∗*: statistically significant.

**Table 4:** Pairwise comparison of skin surface lipids in participants.

Skin surface lipid	P value	Difference	95% Confidence interval
Control vs. mild SSD	0.49	2.91	-2.55 - 8.35
Control vs. moderate SSD	<0.05*∗*	5.65	0.45 - 10.74
Control vs. severe SSD	<0.05*∗*	6.45	0.67 - 12.12
Mild SSD vs. moderate SSD	0.68	2.78	-3.66 - 9.06
Mild SSD vs. severe SSD	0.53	3.56	-3.34 - 10.34
Moderate SSD vs. severe SSD	0.98	0.81	-5.84 - 7.44

*SSD*, scalp seborrheic dermatitis. *∗*: statistically significant.

## Data Availability

The data used to support the findings of this study are available from the corresponding author upon request.
